# Impact of healthcare-associated infections on functional outcome of severe acquired brain injury during inpatient rehabilitation

**DOI:** 10.1038/s41598-022-09351-1

**Published:** 2022-03-28

**Authors:** Michelangelo Bartolo, Chiara Zucchella, Hend Aabid, Beatrice Valoriani, Massimiliano Copetti, Andrea Fontana, Domenico Intiso, Mauro Mancuso

**Affiliations:** 1 Neurorehabilitation Unit, Department of Rehabilitation, HABILITA Zingonia, Via Bologna 1, 24040 Zingonia/Ciserano, BG Italy; 2 Neurology Unit, University Hospital of Verona, Verona, Italy; 3 Medicine Unit, Ospedali Riuniti della Valdichiana, Nottola Hospital, Siena, Italy; 4 Unit of Biostatistics, Fondazione IRCCS Casa Sollievo della Sofferenza, San Giovanni Rotondo, FG Italy; 5 Unit of Neurorehabilitation and Rehabilitation Medicine, Fondazione IRCCS Casa Sollievo della Sofferenza, San Giovanni Rotondo, FG Italy; 6 Physical and Rehabilitative Medicine Unit, NHS-USL Toscana Sud Est, Grosseto, Italy

**Keywords:** Neurology, Neurological disorders, Brain injuries

## Abstract

To describe healthcare-associated infections in inpatient neuro-rehabilitation and their impact on functional outcome, a multicenter observational study with severe acquired brain injury (sABI) patients was performed. Patients were divided into infected (INF-group) or not infected (noINF-group) and assessed at admission and discharge, by means of the Glasgow Coma Scale (GCS), the Rancho Los Amigos Levels of Cognitive Functioning Scale (LCF), the Disability Rating Scale (DRS), and the modified Barthel Index (mBI). One hundred-nineteen patients were included in the INF-group, and 109 in the noINF-group. Culture specimens were found positive for bloodstream (43.8%), respiratory tract (25.7%), urinary tract (16.2%), gastro-intestinal system (8.6%) and skin (2.4%) infections. Multiple microorganisms were the most frequent (58.1%) and 55.5% of patients needed functional isolation due to multidrug resistant germs. The functional status of both groups improved after rehabilitation, but multivariable analyses showed that the INF-group showed a significantly lower gain to GCS (p = 0.008), DRS (p = 0.020) and mBI (p = 0.021) compared to the noINF-group. Length of stay (LOS) and number of skipped rehabilitative sessions were not statistically different between the groups; mortality rate was significantly higher in the INF-group (p = 0.04). Infected sABI patients showed longer LOS, significant increased mortality, and a lower functional outcome than not infected patients.

## Introduction

Hospital-acquired infections or healthcare-associated infections (HAIs) are infections first diagnosed 48 to 72 h after admission to hospital or health care facility^1^. Epidemiological data reveal that, in Europe, about 3.2 million patients develop a HAI every year and that 37.000 subjects die as a direct consequence of HAIs or due to the increasing multidrug resistance (MDR) of HAI-associated pathogens^2^.

Previous studies showed that, in neurological patients, HAIs represent an independent predictor of poor functional outcome and mortality, may delay hospital discharge, with a consequent increase in the costs of care and in the use of medical resources^3–5^. Acute stroke patients with HAIs are less likely to be discharged home and, if post-stroke care is necessary, patients with HAIs are more likely to receive inpatient rehabilitation for lower functional status^6^.

In rehabilitation, favoring factors for HAIs are represented by the high percentage of susceptible patients who need complex therapies, the presence of elderly dependent patients and the use of invasive procedures and of broad-spectrum antibiotics for prophylactic and therapeutic purposes^7,8^. Most of the conditions characterizing the clinical complexity of patients admitted to neurorehabilitation can represent a favoring factor for infections^9^.

Recent studies have reported that HAIs represent a frequent complication for patients affected by severe acquired brain injury (sABI) admitted to intensive neurorehabilitation^10^. Indeed, growing evidence suggests that after acute brain injury, brain-immune interactions may become dysregulated, resulting in the so-called brain-injury induced immunosuppression syndrome, which makes neurological patients more susceptible to developing infections^11^. Furthermore, patients in neurorehabilitation wards have an increased frequency of HAIs due to multidrug resistant (MDR) germs^10,12–14^ which determine additional direct and indirect costs (prolonged hospital stay, increased drugs consumption, necessity of isolation, use of extra laboratory and/or other diagnostic tests) complicating the organizational skills of rehabilitation centers^15^. On this issue, data from the surveillance report by the European Centre for Disease Prevention and Control (ECDC) about the antimicrobial resistance in Europe suggested that the high percentages of resistance to key antimicrobial drugs represent a serious threat to patient safety that need to be addressed by comprehensive infection prevention and control strategies^16^.

In Italy, the frequency of HAIs in hospitalized patients is about 6–10% with a mortality rate of 20–30%^17–19^. So far, to our knowledge no studies have been reported investigating HAIs and functional outcome in sABI patients admitted to the neurorehabilitation setting. A 2-year retrospective cohort study performed to clarify the frequency and the risk factors for infections in patients admitted to 131 rehabilitation units showed that almost 15% of patients acquired at least one infection—the urinary tract infection being the most frequent, followed by respiratory infections^20^. Recently, another study has reported preliminary data on the frequency of hospital-acquired pneumonia in sABI patients but has not provided significant data on functional recovery due to the small sample size^21^. Both studies did not explore the impact of HAIs on the patients’ functional outcome and rehabilitation goals.

As HAIs have the potential for modification, studies exploring the frequency and the impact of infections on patients’ recovery during rehabilitation stay should be pursued in order to develop new preventive strategies. Therefore, the aim of this study was to investigate the frequency and the features of HAIs in sABI patients admitted to intensive neurorehabilitation and to investigate their functional outcome.

## Materials and methods

### Participants

This study enrolled all patients suffering from sABI (Glasgow Coma Scale ≤ 8), consecutively admitted to intensive neurorehabilitation from 1st September 2018 to 28th February 2019. The literature, defines sABI as a central nervous system damage due to acute traumatic or non-traumatic (vascular, anoxic, neoplastic or infective) causes that lead to a variably prolonged state of coma (Glasgow Coma Scale ≤ 8) and a wide range of neurological impairments that affect physical, cognitive and/or psychological functioning^22^.

Exclusion criteria for this study were: previous neurological impairment; infected surgical wounds and/or infected pressure sores at admission in neurorehabilitation; positive infection indexes (e.g.: increase of leukocytes number, erythrocyte sedimentation rate—ESR, C-Reactive Protein—CRP, procalcitonin) at admission in rehabilitation. Subjects with encephalitis were enrolled when the infective process was considered clinically solved and laboratory infection parameters were negative at admission.

The study was a retrospective data analysis, relying on measurements and data collected as part of routine care; therefore, the study was approved by the Institutional Review Board of HABILITA and, according to the national law, notified to the Local Ethics Committee of Bergamo (Italy). The need for informed consent was waived by the Local Ethics Committee of Bergamo, due to the retrospective nature of the study, which was carried out in accordance with the Declaration of Helsinki.

### Study design and procedures

This observational retrospective study was conducted as a part of the standard clinical practice at two neurorehabilitation centers. Patients’ information was entered into the study database anonymously.

Data were collected from medical records and included the following demographic (age, sex) and clinical variables [etiology, lesion site, comorbidities, presence of central venous catheter (CVC), percutaneous endoscopic gastrostomy (PEG), urinary catheter (UC), tracheostomy, pressure sores]. Any surgical or invasive procedure performed during acute care was recorded (ventriculo-peritoneal shunt, craniotomy). The scores obtained from the following clinical scales assessing patients’ clinical and functional status were also archived: the Glasgow Coma Scale (GCS)^23^, The Rancho Los Amigos Levels of Cognitive Functioning Scale (LCFS)^24^, Disability Rating Scale (DRS)^25^, modified Barthel Index (mBI)^26^. The scores obtained within one week from admission (T0) and at discharge (T1) were recorded for each patient. Data about functional isolation, days of interruption of the rehabilitation treatment, mortality and length of stay (LOS) in the rehabilitation setting were also collected. Data about HAIs were recorded and included: frequency, microbial germs, type of culture specimen (hematic, urinary, bronchial and/or tracheostomy secretions, cutaneous, feces, cerebrospinal fluid, CVC), antimicrobial therapy (drugs, time duration).

For the purpose of this study, according to the surveillance definitions of the Centers for Disease Control/National Healthcare Safety Network (CDC/NHSN), patients were defined as “infected” (INF-group) or “not infected” (noINF-group)^27^.

Patients were identified as “infected” when the tissue invasion by microorganisms resulted in a disease, confirmed by fever and blood parameters modification (increase of leukocytes number, ESR, CRP, procalcitonin). In the case of pneumonia, radiological evidence (new or progressive and persistent infiltrates, consolidation or cavitation), increase of leukocytes and inflammatory blood parameters were considered. Patients with signs of infection, in the absence of evidence of pathogens, were considered infected and, if necessary, treated with broad-spectrum antibiotic therapy. When patients presented more than one pathogen species, the infections were categorized as due to multiple pathogens.

Patients who did not exhibit the previous conditions were identified as “not infected” (noINF-group).

### Statistical analysis

Descriptive statistics were reported overall and for the INF-group and the noINF-group, separately, as median and interquartile range for continuous variables and as frequency and percentages for categorical variables. The assumption of normal distribution for each continuous variable was checked by means of the Shapiro–Wilk test. Comparisons between the groups were carried out using the Mann–Whitney U-test and the Pearson chi-square test for continuous and categorical variables, respectively. The Wilcoxon signed-rank test was applied to perform a within-group analysis [admission (T0) − discharge (T1)] with regard to clinical scales.

Multivariable linear analyses were also performed for gain in functional scales adjusting for potential confounders, i.e. functional scale at admission, age, sex, time to admission. As a sensitivity analysis, multivariable linear analyses were further adjusted for etiology.

All tests were 2-sided and the level of statistical significance was set at 0.05. This is an exploratory study without adjusting for multiple testing and that therefore the p-values should be interpreted with caution.

Data processing and statistical analyses were performed with the SPSS Statistics for Windows (version 18.0) (IBM, Armonk NY).

## Results

Two-hundred and twenty-eight consecutive sABI patients [81 females (35.5%), 147 males (64.5%)], with a mean age 65.1 ± 15.3 years were enrolled in the study. The etiologies of sABI were: vascular (n = 121, 53%), encephalitis (n = 10, 4.4%), traumatic (n = 52, 22.8%), neoplastic (n = 4, 1.8%), hypoxic (n = 41, 18%). The mean time from acute brain injury to admission in rehabilitation was 34.9 ± 24.9 days and the mean LOS was 87.1 ± 63.7 days.

Twenty-three patients were excluded from the study because of previous neurological impairment (n = 11), infected pressure sores at admission in rehabilitation (n = 6), positive infection indices at admission in rehabilitation (n = 6).

One hundred and nineteen (52.2%) patients (38 F, 81 M; mean age 66.2 ± 14.1 years) were included in the INF-group, and 109 (47.8%) patients (43 F, 66 M; mean age 64 ± 16.6) in the noINF-group. No statistical differences between the two groups were found for demographic and clinical features, except for a significant higher rate of CVC (p = 0.000) in the INF-group. Functional evaluation was performed in all subjects within a week from admission in rehabilitation: mean 1.8 ± 1.5 days. Demographic and clinical characteristics of the whole sample and of the subgroups (INF-group and noINF-group) are reported in Table 1. These parameters and functional scales have been summarized and reported also for each etiology category in Supplementary Appendix 1.

**Table 1:** Patients’ demographic and clinical features.

	Total sample (n = 228)	INF-group (n = 119)	noINF-group (n = 109)
Age, years, median [25th; 75th quartiles]		69 [59; 76]	67 [54.5; 76]
**Gender *n* (%)**			
Female	81 (35.5)	38 (31.9)	43 (39.4)
Male	147 (64.5)	81 (68.1)	66 (60.6)
**Comorbidities *n* (%)**			
Cardiovascular	85 (37.3)	49 (41.2)	36 (33)
Dysmetabolic/endocrine	9 (3.9)	6 (5)	3 (2.8)
Neoplastic	3 (1.3)	2 (1.7)	1 (0.9)
Psychiatric	3 (1.3)	3 (2.5)	0
Multiple	55 (24.1)	25 (21)	30 (27.5)
Other	6 (2.6)	3 (2.5)	3 (2.8)
**Etiology *n* (%)**			
Ischemic	51 (22.4)	27 (22.7)	24 (22)
Hemorrhagic	70 (30.7)	42 (35.3)	28 (25.7)
Encephalitis	10 (4.4)	7 (5.9)	3 (2.7)
Traumatic	52 (22.8)	20 (16.8)	32 (29.4)
Hypoxic	41 (18)	23 (19.3)	18 (16.5)
Neoplastic	4 (1.7)	0	4 (3.7)
**Lesion site *n* (%)**			
Diffuse damage	53 (23.2)	29 (24.4)	24 (22)
Right hemisphere	51 (22.4)	21 (17.6)	30 (27.5)
Left hemisphere	52 (22.8)	30 (25.2)	22 (20.2)
Bilateral	15 (6.6)	9 (7.6)	6 (5.5)
Posterior cranial fossa	5 (2.2)	4 (3.4)	1 (0.9)
Brainstem	7 (3.1)	1 (0.8)	6 (5.5)
Basal ganglia	15 (6.6)	6 (5)	9 (8.3)
Multiple sites	26 (11.4)	16 (13.4)	10 (9.2)
Other	4 (1.7)	3 (2.6)	1 (0.9)
**Devices *n* (%)**			
Central venous catheter	69 (30.3)	48 (40.3)*	21 (19.3)
Percutaneous endoscopic gastrostomy	93 (40.8)	42 (35.3)	51 (46.8)
Urinary catheter	214 (93.8)	112 (94.1)	102 (93.6)
Tracheostomy	162 (71)	90 (75.6)	72 (66.1)
Ventriculo-peritoneal shunt	13 (5.7)	5 (4.2)	8 (7.3)

Data are expressed as n (%).

*Significance p < 0.05.

No statistical differences between the two groups (INF-group vs noINF-group) were found related to the patients’ unit of provenance: Intensive Care Unit (ICU) (50.4% vs 41.3%), Neurology Unit (13.4% vs 11%), Stroke Unit (10.9% vs 11%), Medicine Unit (11.8% vs 8.3%), Surgery Unit (1.7% vs 1.8%), except for the provenance from the Neurosurgery Unit that showed also a higher rate in the noINF-group (11.8% vs 26.6%; p = 0.007).

### Infections

**Figure 1:**
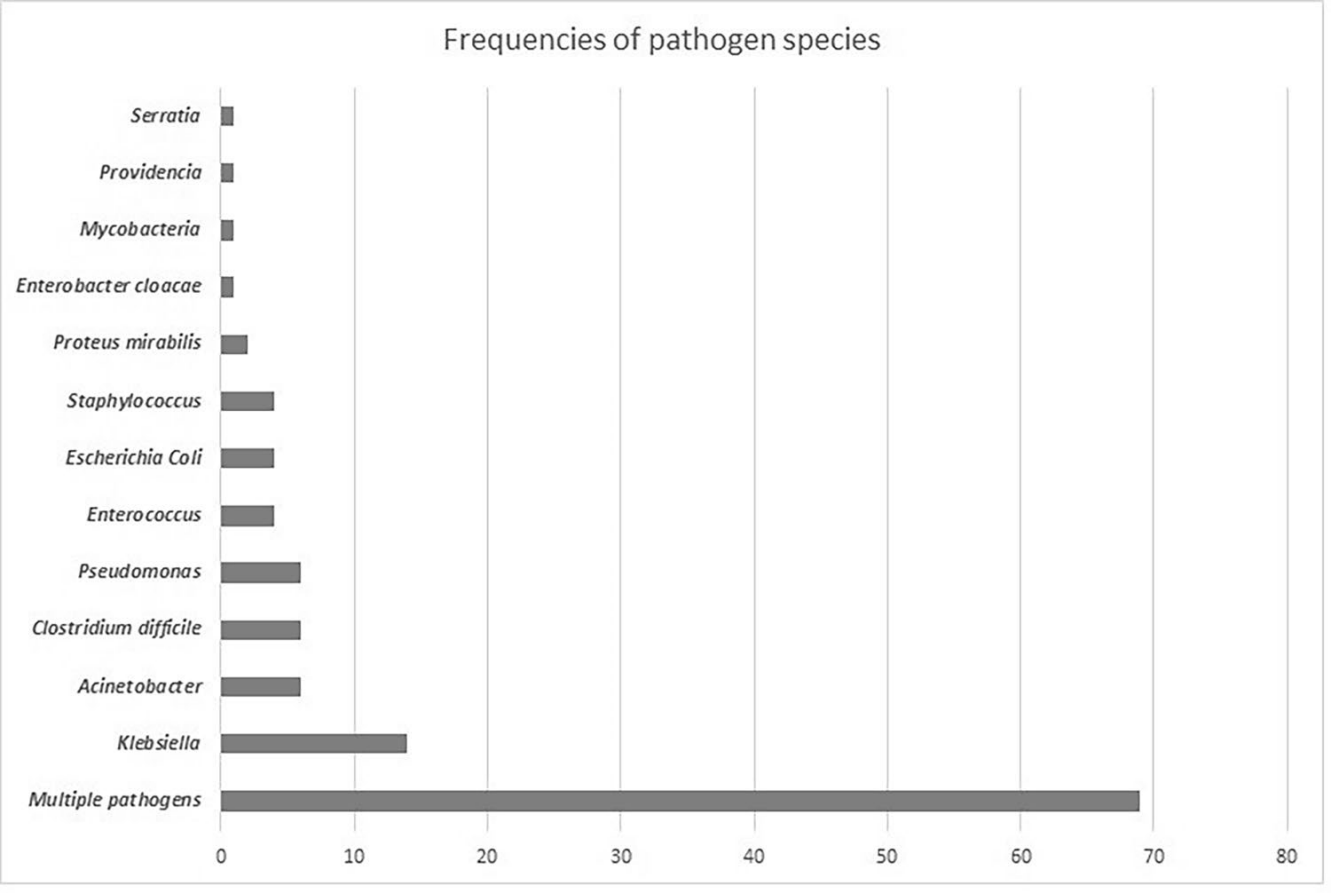
Frequencies of pathogen species.

In the INF-group, 217 culture specimens were taken: 43.4% blood, 16.6% urine, 13.4% expectorated sputum, 8.3% tracheostomy, 8.3% feces, 3.2% CVC, 3.2% oropharyngeal swabs, 2.8% wounds, 0.4% cerebrospinal fluid, and 0.4% vaginal swab. The infections affected several organs and systems including bloodstream 43.8%, respiratory tract 25.7% (pneumonia 16.6%, upper airways 9.0%), urinary tract 16.2%, gastro-intestinal tract 8.6%, and skin wounds 2.4%. Furthermore, infected devices were detected: catheter-related infections 3.3%. The relative frequencies (percentage) of the different microbial germs are reported in Fig. 1.

The presence of multiple microorganisms species was by far the most frequent (n = 69, 58.1%). In the INF-group, 66 (55.5%) patients need functional isolation due to MDR germs, in particular: *Acinetobacter baumannii*, Enterobacteriaceae, *Pseudomonas aeruginosa*, *Staphilococcus aureus*. Patients in the INF-group were administered antibiotic therapy for an average time of 20.1 ± 11.4 days. Forty-eight (40.3%) patients took antibiotics as monotherapy, including: 13 (10.9%) cephalosporins, 9 (7.6%) carbapenemics, 7 (5.9%) rifamycins, 5 (4.2%) glycopeptides, 4 (3.4%) quinolone, 4 (3.4%) aminoglycoside, 2 (1.7%) colistin or tigecycline, 1 (0.8%) penicillinic drugs, 1 (0.8%) nitroimidazole. Seventy-one 71 (59.7%) patients took multiple antibiotics.

### Functional outcome

The comparison between the functional scales scores from the two groups showed that the INF-group had a significantly higher disability at admission than the noINF-group (Table 2). After rehabilitation, both groups showed an improvement in all scales. In particular, the noINF-group showed a significant improvement in GCS (p = 0.03), LCF (p = 0.0001), DRS (p < 0.0001) and mBI (p < 0.001); whereas the INF-group had a significant improvement only in DRS (p < 0.0001) and mBI (p < 0.01). GCS and LCF score increased, but not significantly, after rehabilitation in the INF-group: p = 0.51 and p = 0.6, respectively. With regard to GCS, in the INF-group, 26 (21.8%) patients were able to follow commands at admission, 35 (29.4%) at discharge; in the no-INF-group 49 (44.9%) patients were able to follow commands at admission, 68 (62.4%) at discharge.

**Table 2:** Outcome measures at admission and discharge from rehabilitation in all patients, INF-group and noINF-group.

Outcome measures	Total sample	INF-group	noINF-group	*p* value
**GCS**				
Admission	10.00 [6;14]	8 [6; 11]	12 [8; 14]	0.000
Discharge	12.00 (5.75, 14.00)	11.5 [7.75; 14]	14 [11; 15]	0.001
Change	0.00 (0.00, 2.00)	1 [0; 3]	0.5 [0; 2]	0.138
**LCF**				
Admission	3.00 (2.00, 4.00)	3 [2; 4]	3 [2; 5]	0.001
Discharge	4.00 (2.00, 6.00)	4 [2; 5.5]	5 [3; 6]	0.016
Change	1.00 (0.00, 1.25)	1 [0; 2]	1 [0; 2]	0.015
**DRS**				
Admission	9.00 (7.00, 21.00)	19.5 [9; 25]	8 [7; 14.25]	0.000
Discharge	7.00 (3.00, 11.00)	12 [8; 20]	6 [4; 9]	0.000
Change	− 2.00 (− 6.00, 0.00)	− 1 [− 4.25; 0]	− 2 [− 4; 0]	0.058
**mBI**				
Admission	0.00 (0.00, 0.00)	0 [0; 0]	0 [0; 8]	0.000
Discharge	0.00 (0.00, 20.00)	0 [0; 10]	15 [0; 70]	0.000
Change	0.00 (0.00, 15.00)	8 [0; 8.25]	8.5 [0; 34.75]	0.001

Between group analysis. Data are expressed as median and interquartile range IQR [25; 75].

*GCS* Glasgow Coma Scale, *LCFS* The Rancho Los Amigos Levels of Cognitive Functioning Scale, *DRS* Disability Rating Scale, *mBI* modified Barthel Index.

In the univariate analyses, between-group comparisons of gain calculated for each scale showed that the noINF-group had a statistically higher gain only in LCF and the mBI (Table 2). The multivariate analyses adjusted for functional scales at admission, age, sex and time to admission showed that the INF-group had a significantly lower gain in GCS (p = 0.008), DRS (p = 0.020) and mBI (p = 0.021) with respect to the noINF-group (Table 3). On the other hand, gain in LCF was no longer significant. When a further adjustment for etiology was included in the multivariable analyses, only the gain in CGS (p = 0.021) resulted significantly lower in the INF-group with respect to the not infected group (Table II Appendix on line).

**Table 3:** Gain in functional scales in the INF-group compared to the NoINF-group. Multivariable linear analyses.

	GCS gain			LCF gain			DRS gain			mBI gain		
	Estimate	Std. error	p value	Estimate	Std. error	p value	Estimate	Std. error	p value	Estimate	Std. error	p value
(Intercept)	3.23	0.95	0.0009	1.21	0.61	0.0488	− 1.04	0.93	0.2635	12.49	10.49	0.2364
**Group (INF vs NoINF)**	− 1.09	0.40	0.0081	− 0.43	0.27	0.1177	0.68	0.29	0.0201	− 11.49	4.93	0.0217
Functional scale at admission	− 0.18	0.05	0.0015	0.06	0.08	0.4532	0.02	0.08	0.8404	0.19	0.12	0.1356
Age (years)	0.003	0.01	0.7886	− 0.004	0.01	0.6235	− 0.002	0.01	0.7819	0.09	0.15	0.5662
Gender (F vs. M)	0.07	0.38	0.8518	− 0.13	0.26	0.6050	− 0.42	0.27	0.1297	3.36	4.78	0.4837
Time to admission (days)	− 0.001	0.00	0.2900	0.0001	0.00	0.8662	0.0002	0.00	0.7354	0.004	0.01	0.6174

*INF* infected group, *NoINF* not infected group, *F* female, *M* male, *GCS* Glasgow Coma Scale, *LCF* The Rancho Los Amigos Level of Cognitive Functioning, *DRS* Disability Rating Scale, *mBI* modified Barthel Index.

Patients in the INF-group skipped an average number of 0.67 ± 1.51 rehabilitation sessions; such number is not significantly different from the one registered for the noINF-group (0.45 ± 1.15 session). Although the INF-group showed a higher absolute value, no significant difference was found in the LOS (INF-group 90.7 ± 56.8 days vs noINF-group 83.2 ± 70.6 days).

During the rehabilitation stay, a mortality rate of 21.9% (50 patients) was globally detected. However, the INF-group showed a significant higher mortality rate than the noINF-group (27.7%, 33 patients vs 15.6%, 17 patients; p = 0.04).

## Discussion

The results of this study show that more than a half of sABI patients developed HAIs during their neurorehabilitation stay. The infected patients showed a higher level of functional impairment at admission and achieved a significantly lower gain in all functional scale scores, except LCF, than the not infected patients. However, despite this negative functional picture, the infected sABI patients benefited from the intensive rehabilitation intervention. Infections were predominantly due to multiple pathogen germs and required three weeks of antibiotic therapy on average.

The frequency of infections detected in the present study is dramatically higher compared to that reported in previous studies^21^. However, the finding is not surprising for several reasons. It is known that brain injuries can induce a modulation of the immunologic response from a transitory activation to a systemic immunodepression that can persist for several weeks^28^, making sABI patients more prone to developing infections. Moreover, it must also be considered that sABI patients admitted to neurorehabilitation come from acute care settings, where the use of medical devices (CVC, UC, tracheostomy) is frequent and, despite the adoption of precautionary strategies, they may be carriers of infections^29^.

Furthermore, since the limitation of resources and organizational needs, there is an increasing pressure by acute care settings (i.e. Intensive Care Units, Neurosurgery, Stroke Units,…) in order to speed up patient transfer to neurorehabilitation when the clinical conditions are almost completely stable and latent or manifest infections could be ongoing. On this topic, a recent paper has showed that, in sABI patients’ persistent infections and sepsis during rehabilitation were the main causes of readmission to the ICU^30^.

Like previous studies, also in this study the infections concerned predominantly bloodstream, respiratory and urinary systems^31^. In our opinion, the presence of multiple medical devices, as far as the prolonged (often obliged) bed or supine position, represent potential access doors to the organism and factors reducing the respiratory performance that globally negatively impact on the breathing performances and ultimately promote the onset and/or the development of infections.

Regarding functional recovery and outcomes, although both groups achieved improvement in the clinical and functional scales after rehabilitation, the INF-group obtained a significant lower functional recovery than the not infected patients. The multivariate analyses adjusted for age, sex and time to admission confirmed that the INF-group had a significantly lower improvement in all functional scales except LCF. However, when adjustment for etiology was included in the multivariable analyses, only the gain in CGS (p = 0.021) resulted significantly lower in the INF-group. It should not be forgotten that the other effects in the sensitivity analysis, although not statistically significant, remained in the same direction. Although the small size of subgroups could limit the finding, the etiology of sABI might affect the outcome and should be considered in future studies.

A relevant finding of the present study is that among the infected patients, functional isolation was necessary in up to 55.5% of patients. In subjects who undergo rehabilitation, this organizational measure raises several questions relating to the need for a dedicated setting and care with related costs, to the effect of isolation on the outcome as well as to the number and type of delivered rehabilitative interventions. Furthermore, some ethical concerns have to be considered. In fact, according to previous studies, isolation measures may cause psychological distress symptoms including depression and anxiety in patients and relatives^32^. In this study, since no difference was detected between the two groups in the number of rehabilitative sessions, the poorest outcome observed in the INF-group cannot be attributed to a lower number of treatments. Moreover, even if patient isolation was required for more than half of the infected patients, mobilization in bed and/or physical exercises were provided in the room. Consistently, two recent studies have investigated the impact of contact precautions on the outcome of neurological patients during early rehabilitation, showing that functional recovery of patients infected or colonized with multidrug resistant bacteria was poorer than in the not infected patients. However, the authors concluded that in patients who underwent isolation, the poorer outcome was not due to a reduced rehabilitation therapy but to lower functional status and higher morbidity upon admission^13,33^. In our view, this finding could be attributable to organizational features of neurorehabilitation wards that usually include dedicated beds in managing sABI patients and to technological supports (motorized beds, robotic tilt tables, and so on.) in order to allow easier rehabilitation treatments, also in patients with worse functional conditions. Anyway, what should be the best care management of subjects who need isolation but requiring rehabilitation remains unclear. In this respect, a recent survey has showed that, at the moment, in Europe a widely accepted consensus on how patients with MDR infections should be managed in a rehabilitation setting is still lacking^34^.

Although no statistically significantdifferences were found between the two groups, LOS was higher in the infected sABI patients. Likewise, a significantly higher mortality rate was detected in this group. Therefore, HAI in the sABI patients is a complication that can worsen the clinical picture, hinder the recovery and delay the discharge.

Although significant progress has been made with regard to the implementation of the best practices for the prevention of HAIs, efforts must be made to reduce their frequency.

The World Health Organization (W.H.O.) recommends Healthcare Systems to develop actions useful to promote the reduction of HAIs, through the engagement between public health agencies, healthcare professionals and local institutions for the implementation, sustainability and expansion of programs for the surveillance and prevention of HAIs. A successful strategy in the control of HAI refers to the adoption of interventions or best-practice bundles. Monitoring the adherence to best practices, education, and establishment of process, structure and outcome indicators are essential for further reducing its incidence. These actions should be also implemented in the neurorehabilitation setting to improve the quality of care^35^.

Antibiotic exposure is well recognized as the most important modifiable risk factor for CDI, and antibiotic stewardship is potentially the most effective CDI prevention strategy^36,37^. According to the findings of the present study and previous literature, the implementation of hospital infections and antibiotic stewardship programs are needed to manage HAIs prevention^36^.

Some limitations of this work need to be pointed out: first of all, the local nature of the experience which does not permit extrapolation and generalization from these findings as well as the retrospective design of the study (reliance on accuracy of written records). Moreover, none of the infections that occurred before rehabilitation were considered and, therefore, the effects of the infections from the acute phase could have been neglected. However, in our opinion, this study can help to focus on the study question, clarify the hypothesis and identify feasibility issues for future prospective studies. In fact, several issues about the best care management of sABI subjects with HAIs remain unsolved, particularly in those patients with MDR infections; also, proper investigations should be planned. Furthermore, whether the site of infections, type of germs and etiology of sABI may influence the outcome, remains unclear. Meanwhile, preventing HAIs and the consequent spread of antibiotic resistance is possible if physicians, nurses and healthcare leaders consistently and comprehensively follow all recommendations to prevent HAIs, including hand hygiene, room cleaning, use of personal protective equipment, prevention of catheter- and procedure-related infections, antimicrobial stewardship, and implementation of measures to prevent spread.

In conclusion, beyond sABI etiology that represents a strong predictor of functional outcome, also HAIs impact on the rehabilitation pathway of the sABI patients, determining extended LOS, a significantly higher mortality and a poorer outcome than the subjects without HAI. Since many hospital-acquired conditions can be prevented^38,39^, also considering the magnitude of the clinical and economic burden of HAIs, the current emphasis on implementing interventions aiming to decrease the incidence of HAIs may have a potentially large impact.

## Supplementary Information

Supplementary Information 1.

Supplementary Information 2.
