# Supplementary material for: Impact of healthcare-associated infections on functional outcome of severe acquired brain injury during inpatient rehabilitation
Source: Sci Rep. 2022 Mar 28;12:5245. doi: 10.1038/s41598-022-09351-1 (PMC8960831; doi:10.1038/s41598-022-09351-1)
Supplement: Supplementary file 1 — Supplementary Information 1. [file 41598_2022_9351_MOESM1_ESM.doc]

Appendix 1. Demographics and functional scales for etiology in sABI.

|  | Ischemic (N=49) | Haemorrhagic (N=70) | Encephalitis (N=10) | Traumatic (N=52) | Neoplastic (N=4) | Hypoxic  (N=41) | Total  (N=226) | p value |
| --- | --- | --- | --- | --- | --- | --- | --- | --- |
| **Age** |  |  |  |  |  |  |  | 0.459 |
| Mean (SD) | 68.92 (13.49) | 65.69 (9.70) | 59.70 (25.73) | 63.13 (18.99) | 60.25 (14.17) | 64.32 (17.18) | 65.19 (15.40) |  |
| Median (Q1, Q3) | 72.00 (61.00, 80.00) | 68.00 (57.25, 72.75) | 65.00 (55.50, 74.50) | 69.00 (55.25, 77.25) | 57.50 (50.75, 67.00) | 66.00 (56.00, 76.00) | 68.50 (56.25, 76.00) |  |
| Min - Max | 30.00 - 89.00 | 43.00 - 84.00 | 6.00 - 92.00 | 19.00 - 85.00 | 47.00 - 79.00 | 18.00 - 86.00 | 6.00 - 92.00 |  |
| Non missing N | 49 | 70 | 10 | 52 | 4 | 41 | 226 |  |
| **Sex** |  |  |  |  |  |  |  | 0.138 |
| Mean (SD) | 1.73 (0.45) | 1.56 (0.50) | 1.40 (0.52) | 1.65 (0.48) | 1.50 (0.58) | 1.73 (0.45) | 1.64 (0.48) |  |
| Median (Q1, Q3) | 2.00 (1.00, 2.00) | 2.00 (1.00, 2.00) | 1.00 (1.00, 2.00) | 2.00 (1.00, 2.00) | 1.50 (1.00, 2.00) | 2.00 (1.00, 2.00) | 2.00 (1.00, 2.00) |  |
| Min - Max | 1.00 - 2.00 | 1.00 - 2.00 | 1.00 - 2.00 | 1.00 - 2.00 | 1.00 - 2.00 | 1.00 - 2.00 | 1.00 - 2.00 |  |
| Non missing N | 49 | 70 | 10 | 52 | 4 | 41 | 226 |  |
| **LOS** |  |  |  |  |  |  |  | 0.065 |
| Mean (SD) | 75.65 (56.38) | 99.53 (61.88) | 67.90 (52.57) | 79.85 (67.77) | 45.00 (36.18) | 99.20 (70.82) | 87.40 (63.85) |  |
| Median (Q1, Q3) | 58.00 (30.00, 92.00) | 74.00 (53.25, 157.25) | 56.00 (34.00, 93.50) | 60.50 (27.00, 112.75) | 32.00 (24.75, 52.25) | 84.00 (33.00, 177.00) | 65.00 (35.00, 136.75) |  |
| Min - Max | 7.00 - 213.00 | 2.00 - 278.00 | 4.00 - 183.00 | 10.00 - 355.00 | 18.00 - 98.00 | 3.00 - 244.00 | 2.00 - 355.00 |  |
| Non missing N | 49 | 70 | 10 | 52 | 4 | 41 | 226 |  |
| **Time to admission** |  |  |  |  |  |  |  | < 0.001 |
| Mean (SD) | 60.71 (137.02) | 58.52 (78.35) | 78.80 (63.95) | 224.56 (545.65) | 27.50 (29.86) | 77.73 (60.62) | 96.79 (270.57) |  |
| Median (Q1, Q3) | 15.00 (13.00, 27.00) | 35.00 (18.00, 58.25) | 61.00 (41.00, 77.00) | 38.00 (26.00, 111.00) | 16.00 (8.50, 35.00) | 60.00 (40.50, 81.00) | 35.00 (18.00, 67.00) |  |
| Min - Max | 1.00 - 647.00 | 9.00 - 424.00 | 27.00 - 188.00 | 13.00 - 2399.00 | 7.00 - 71.00 | 19.00 - 232.00 | 1.00 - 2399.00 |  |
| Non missing N | 28 | 40 | 5 | 25 | 4 | 15 | 117 |  |
| Missing N | 21 | 30 | 5 | 27 | 0 | 26 | 109 |  |
| **INF-group** |  |  |  |  |  |  |  | 0.040 |
| Mean (SD) | 0.55 (0.50) | 0.60 (0.49) | 0.70 (0.48) | 0.38 (0.49) | 0.00 (0.00) | 0.56 (0.50) | 0.53 (0.50) |  |
| Median (Q1, Q3) | 1.00 (0.00, 1.00) | 1.00 (0.00, 1.00) | 1.00 (0.25, 1.00) | 0.00 (0.00, 1.00) | 0.00 (0.00, 0.00) | 1.00 (0.00, 1.00) | 1.00 (0.00, 1.00) |  |
| Min - Max | 0.00 - 1.00 | 0.00 - 1.00 | 0.00 - 1.00 | 0.00 - 1.00 | 0.00 - 0.00 | 0.00 - 1.00 | 0.00 - 1.00 |  |
| Non missing N | 49 | 70 | 10 | 52 | 4 | 41 | 226 |  |
| **GCS (admission)** |  |  |  |  |  |  |  | < 0.001 |
| Mean (SD) | 11.39 (3.60) | 8.40 (3.48) | 9.20 (4.32) | 9.79 (3.96) | 13.25 (2.22) | 8.75 (4.10) | 9.56 (3.92) |  |
| Median (Q1, Q3) | 13.00 (10.00, 14.00) | 8.00 (6.00, 11.00) | 9.00 (5.25, 13.75) | 10.50 (6.00, 13.00) | 14.00 (13.00, 14.25) | 8.00 (6.00, 12.50) | 10.00 (6.00, 14.00) |  |
| Min - Max | 3.00 - 15.00 | 3.00 - 15.00 | 3.00 - 14.00 | 3.00 - 15.00 | 10.00 - 15.00 | 2.00 - 15.00 | 2.00 - 15.00 |  |
| Non missing N | 49 | 70 | 10 | 52 | 4 | 40 | 225 |  |
| Missing N | 0 | 0 | 0 | 0 | 0 | 1 | 1 |  |
| **GCS (discharge)** |  |  |  |  |  |  |  | 0.002 |
| Mean (SD) | 11.06 (5.26) | 8.81 (5.23) | 7.80 (6.46) | 10.23 (5.62) | 14.50 (1.00) | 7.34 (5.44) | 9.42 (5.53) |  |
| Median (Q1, Q3) | 13.00 (10.00, 15.00) | 10.00 (5.25, 13.00) | 7.50 (1.25, 14.00) | 13.00 (6.00, 15.00) | 15.00 (14.50, 15.00) | 6.00 (3.00, 12.00) | 12.00 (6.00, 14.00) |  |
| Min - Max | 0.00 - 15.00 | 0.00 - 15.00 | 0.00 - 15.00 | 0.00 - 15.00 | 13.00 - 15.00 | 0.00 - 15.00 | 0.00 - 15.00 |  |
| Non missing N | 49 | 70 | 10 | 52 | 4 | 41 | 226 |  |
| **GGS (change)** |  |  |  |  |  |  |  | 0.018 |
| Mean (SD) | -0.33 (4.31) | 0.41 (4.89) | -1.40 (2.63) | 0.44 (4.57) | 1.25 (1.26) | -1.20 (4.27) | -0.10 (4.48) |  |
| Median (Q1, Q3) | 0.00 (0.00, 1.00) | 1.00 (0.00, 3.00) | 0.00 (-3.00, 0.75) | 0.00 (0.00, 3.00) | 1.00 (0.75, 1.50) | 0.00 (-1.00, 0.00) | 0.00 (0.00, 2.00) |  |
| Min - Max | -15.00 - 10.00 | -15.00 - 9.00 | -6.00 - 1.00 | -12.00 - 11.00 | 0.00 - 3.00 | -15.00 - 7.00 | -15.00 - 11.00 |  |
| Non missing N | 49 | 70 | 10 | 52 | 4 | 41 | 226 |  |
| **LCF (admission)** |  |  |  |  |  |  |  | < 0.001 |
| Mean (SD) | 4.06 (1.57) | 2.71 (1.26) | 3.10 (0.99) | 3.31 (1.59) | 5.50 (1.73) | 3.00 (1.70) | 3.26 (1.59) |  |
| Median (Q1, Q3) | 4.00 (3.00, 5.00) | 2.00 (2.00, 3.00) | 3.00 (2.25, 3.75) | 3.00 (2.00, 4.25) | 6.00 (5.25, 6.25) | 2.00 (2.00, 4.00) | 3.00 (2.00, 4.00) |  |
| Min - Max | 2.00 - 8.00 | 1.00 - 6.00 | 2.00 - 5.00 | 1.00 - 6.00 | 3.00 - 7.00 | 1.00 - 7.00 | 1.00 - 8.00 |  |
| Non missing N | 49 | 70 | 10 | 52 | 4 | 41 | 226 |  |
| **LCF (discharge)** |  |  |  |  |  |  |  | 0.002 |
| Mean (SD) | 4.42 (2.35) | 3.33 (2.27) | 2.60 (2.32) | 3.98 (2.49) | 6.25 (2.22) | 2.83 (2.28) | 3.64 (2.42) |  |
| Median (Q1, Q3) | 5.00 (3.00, 6.00) | 3.00 (2.00, 5.00) | 2.50 (0.50, 3.75) | 5.00 (2.00, 6.00) | 7.00 (6.00, 7.25) | 2.00 (1.00, 4.00) | 4.00 (2.00, 6.00) |  |
| Min - Max | 0.00 - 8.00 | 0.00 - 8.00 | 0.00 - 7.00 | 0.00 - 8.00 | 3.00 - 8.00 | 0.00 - 7.00 | 0.00 - 8.00 |  |
| Non missing N | 48 | 69 | 10 | 51 | 4 | 41 | 223 |  |
| Missing N | 1 | 1 | 0 | 1 | 0 | 0 | 3 |  |
| **LCF (change)** |  |  |  |  |  |  |  | 0.083 |
| Mean (SD) | 0.27 (2.12) | 0.57 (1.93) | -0.50 (1.65) | 0.60 (2.12) | 0.75 (0.50) | -0.17 (1.82) | 0.33 (1.98) |  |
| Median (Q1, Q3) | 1.00 (0.00, 1.00) | 1.00 (0.00, 2.00) | 0.00 (-1.50, 0.00) | 1.00 (0.00, 2.00) | 1.00 (0.75, 1.00) | 0.00 (0.00, 1.00) | 1.00 (0.00, 1.75) |  |
| Min - Max | -8.00 - 3.00 | -4.00 - 6.00 | -3.00 - 2.00 | -5.00 - 5.00 | 0.00 - 1.00 | -5.00 - 3.00 | -8.00 - 6.00 |  |
| Non missing N | 49 | 70 | 10 | 52 | 4 | 41 | 226 |  |
| **DRS (admission)** |  |  |  |  |  |  |  | 0.008 |
| Mean (SD) | 12.96 (8.18) | 14.25 (8.27) | 14.90 (8.20) | 14.40 (7.93) | 5.75 (1.26) | 15.72 (7.82) | 14.14 (8.07) |  |
| Median (Q1, Q3) | 8.00 (6.00, 20.00) | 9.00 (8.00, 22.00) | 14.00 (7.50, 22.25) | 10.00 (7.75, 21.00) | 6.00 (5.50, 6.25) | 16.00 (9.00, 21.50) | 9.00 (7.00, 21.00) |  |
| Min - Max | 3.00 - 29.00 | 2.00 - 29.00 | 6.00 - 26.00 | 4.00 - 29.00 | 4.00 - 7.00 | 1.00 - 29.00 | 1.00 - 29.00 |  |
| Non missing N | 49 | 69 | 10 | 52 | 4 | 40 | 224 |  |
| Missing N | 0 | 1 | 0 | 0 | 0 | 1 | 2 |  |
| **DRS (discharge)** |  |  |  |  |  |  |  | 0.628 |
| Mean (SD) | 8.86 (7.91) | 9.13 (7.22) | 7.10 (6.61) | 7.88 (6.34) | 4.50 (1.73) | 9.55 (8.70) | 8.69 (7.38) |  |
| Median (Q1, Q3) | 6.00 (3.00, 15.00) | 7.50 (5.00, 10.00) | 6.50 (1.25, 9.00) | 7.00 (3.00, 10.00) | 4.00 (3.75, 4.75) | 9.00 (0.75, 12.25) | 7.00 (3.00, 11.00) |  |
| Min - Max | 0.00 - 29.00 | 0.00 - 28.00 | 0.00 - 20.00 | 0.00 - 24.00 | 3.00 - 7.00 | 0.00 - 28.00 | 0.00 - 29.00 |  |
| Non missing N | 49 | 68 | 10 | 50 | 4 | 40 | 221 |  |
| Missing N | 0 | 2 | 0 | 2 | 0 | 1 | 5 |  |
| **DRS (change)** |  |  |  |  |  |  |  | 0.554 |
| Mean (SD) | -4.10 (6.72) | -5.17 (8.32) | -7.80 (11.50) | -6.83 (8.91) | -1.25 (1.50) | -6.02 (8.64) | -5.52 (8.30) |  |
| Median (Q1, Q3) | -1.00 (-4.00, 0.00) | -1.00 (-5.00, 0.00) | -0.50 (-18.25, 0.00) | -3.00 (-10.00, -0.75) | -1.00 (-2.25, 0.00) | 0.00 (-11.00, 0.00) | -2.00 (-6.00, 0.00) |  |
| Min - Max | -26.00 - 3.00 | -29.00 - 4.00 | -26.00 - 0.00 | -29.00 - 1.00 | -3.00 - 0.00 | -29.00 - 0.00 | -29.00 - 4.00 |  |
| Non missing N | 49 | 70 | 10 | 52 | 4 | 41 | 226 |  |
| **mBI (admis.)** |  |  |  |  |  |  |  | 0.001 |
| Mean (SD) | 11.40 (21.67) | 4.46 (15.87) | 8.70 (14.21) | 5.04 (12.58) | 16.75 (19.17) | 0.98 (3.57) | 5.85 (15.56) |  |
| Median (Q1, Q3) | 0.00 (0.00, 8.25) | 0.00 (0.00, 0.00) | 1.50 (0.00, 8.75) | 0.00 (0.00, 0.00) | 11.50 (6.00, 22.25) | 0.00 (0.00, 0.00) | 0.00 (0.00, 0.00) |  |
| Min - Max | 0.00 - 84.00 | 0.00 - 89.00 | 0.00 - 40.00 | 0.00 - 60.00 | 0.00 - 44.00 | 0.00 - 20.00 | 0.00 - 89.00 |  |
| Non missing N | 48 | 70 | 10 | 51 | 4 | 41 | 224 |  |
| Missing N | 1 | 0 | 0 | 1 | 0 | 0 | 2 |  |
| **mBI (disch.)** |  |  |  |  |  |  |  | 0.002 |
| Mean (SD) | 27.96 (34.52) | 10.69 (23.40) | 15.80 (27.36) | 24.59 (32.98) | 56.25 (37.50) | 5.97 (14.01) | 17.78 (29.19) |  |
| Median (Q1, Q3) | 7.50 (0.00, 68.50) | 0.00 (0.00, 7.25) | 1.50 (0.00, 13.75) | 2.00 (0.00, 40.00) | 75.00 (56.25, 75.00) | 0.00 (0.00, 1.25) | 0.00 (0.00, 20.00) |  |
| Min - Max | 0.00 - 90.00 | 0.00 - 96.00 | 0.00 - 80.00 | 0.00 - 94.00 | 0.00 - 75.00 | 0.00 - 60.00 | 0.00 - 96.00 |  |
| Non missing N | 48 | 70 | 10 | 51 | 4 | 40 | 223 |  |
| Missing N | 1 | 0 | 0 | 1 | 0 | 1 | 3 |  |
| **mBI (change)** |  |  |  |  |  |  |  | 0.002 |
| Mean (SD) | 16.22 (23.45) | 6.23 (13.14) | 7.10 (15.80) | 19.17 (28.30) | 39.50 (30.60) | 4.85 (12.56) | 11.75 (21.27) |  |
| Median (Q1, Q3) | 1.00 (0.00, 25.00) | 0.00 (0.00, 5.00) | 0.00 (0.00, 5.00) | 0.00 (0.00, 30.50) | 45.50 (23.25, 61.75) | 0.00 (0.00, 0.00) | 0.00 (0.00, 15.00) |  |
| Min - Max | 0.00 - 85.00 | 0.00 - 60.00 | 0.00 - 51.00 | 0.00 - 92.00 | 0.00 - 67.00 | 0.00 - 60.00 | 0.00 - 92.00 |  |
| Non missing N | 49 | 70 | 10 | 52 | 4 | 41 | 226 |  |

Legend: sABI = severe acquired brain injury; LOS = Length of stay in rehabilitation; GCS = Glasgow Coma Scale; LCF = The Rancho Los Amigos Level of Cognitive Functioning; DRS = Disability rating scale
