# Supplementary material for: Impact of healthcare-associated infections on functional outcome of severe acquired brain injury during inpatient rehabilitation
Source: Sci Rep. 2022 Mar 28;12:5245. doi: 10.1038/s41598-022-09351-1 (PMC8960831; doi:10.1038/s41598-022-09351-1)
Supplement: Supplementary file 2 — Supplementary Information 2. [file 41598_2022_9351_MOESM2_ESM.doc]

Appendix II. The multivariable analyses also adjusted for etiology in sABI.

|  | **GCS gain** | | | **LCF gain** | | | **DRS gain** | | | **mBarthel Index gain** | | |
| --- | --- | --- | --- | --- | --- | --- | --- | --- | --- | --- | --- | --- |
|  | Estimate | Std. Error | P value | Estimate | Std. Error | P value | Estimate | Std. Error | P value | Estimate | Std. Error | P value |
| (Intercept) | 4.05 | 1.24 | 0.0015 | 1.43 | 0.79 | 0.0726 | -1.23 | 0.97 | 0.2071 | 22.05 | 12.14 | 0.0722 |
| **group (INF vs NonINF)** | -0.93 | 0.40 | 0.0212 | -0.32 | 0.27 | 0.2428 | 0.53 | 0.29 | 0.0671 | -7.66 | 4.77 | 0.1113 |
| functional scale at admission | -0.21 | 0.06 | 0.0013 | 0.05 | 0.09 | 0.5866 | -0.01 | 0.09 | 0.8794 | 0.08 | 0.12 | 0.4992 |
| age (years) | -0.01 | 0.01 | 0.6400 | -0.01 | 0.01 | 0.3427 | 0.002 | 0.01 | 0.8528 | 0.05 | 0.15 | 0.7484 |
| gender (f vs. m) | 0.16 | 0.38 | 0.6793 | -0.14 | 0.26 | 0.5893 | -0.44 | 0.27 | 0.1058 | 4.62 | 4.62 | 0.3197 |
| Ischemic | Reference class |  |  | Reference class |  |  | Reference class |  |  | Reference class |  |  |
| Haemorrhagic | 0.45 | 0.54 | 0.4097 | 0.22 | 0.36 | 0.5405 | 0.22 | 0.38 | 0.5661 | -15.50 | 6.01 | 0.0112 |
| Encephalitis | -1.49 | 1.00 | 0.1389 | -0.94 | 0.70 | 0.1813 | 0.78 | 0.73 | 0.2879 | -10.26 | 12.05 | 0.3963 |
| Traumatic | 0.43 | 0.60 | 0.4730 | 0.54 | 0.41 | 0.1889 | -0.34 | 0.43 | 0.4218 | 2.89 | 7.06 | 0.6832 |
| Neoplastic | 0.24 | 1.05 | 0.8237 | -0.39 | 0.73 | 0.5918 | 0.16 | 0.77 | 0.8333 | 10.81 | 12.76 | 0.3990 |
| Hypoxic | -1.70 | 0.75 | 0.0250 | -0.68 | 0.51 | 0.1810 | 1.15 | 0.50 | 0.0221 | -24.03 | 8.27 | 0.0045 |
| time to admission (days) | 0.00 | 0.00 | 0.1808 | 0.00 | 0.00 | 0.7507 | 0.00 | 0.00 | 0.4347 | 0.00 | 0.01 | 0.9473 |

Legend: sABI = severe acquired brain injury; LOS = Length of stay in rehabilitation; GCS = Glasgow Coma Scale; LCF = The Rancho Los Amigos Level of Cognitive Functioning; DRS = Disability rating scale
